# Predicting breast cancer metastasis from whole-blood transcriptomic measurements

**DOI:** 10.1186/s13104-020-05088-0

**Published:** 2020-05-20

**Authors:** Einar Holsbø, Vittorio Perduca, Lars Ailo Bongo, Eiliv Lund, Etienne Birmelé

**Affiliations:** 1 Department of Computer Science, UiT – The Arctic University of Norway, Tromsø, Norway; 2 Laboratoire MAP5 (UMR CNRS 8145), Université Paris Descartes, Université de Paris, Paris, France; 3 Cancer Registry of Norway, Oslo, Norway; 4 Department of Community Medicine, UiT – The Arctic University of Norway, Tromsø, Norway

**Keywords:** Transcriptomics, Predictive models, Metastasis, Breast cancer, Epidemiology

## Abstract

**Objective:**

In this exploratory work we investigate whether blood gene expression measurements predict breast cancer metastasis. Early detection of increased metastatic risk could potentially be life-saving. Our data comes from the Norwegian Women and Cancer epidemiological cohort study. The women who contributed to these data provided a blood sample up to a year before receiving a breast cancer diagnosis. We estimate a penalized maximum likelihood logistic regression. We evaluate this in terms of calibration, concordance probability, and stability, all of which we estimate by the bootstrap.

**Results:**

We identify a set of 108 candidate predictor genes that exhibit a fold change in average metastasized observation where there is none for the average non-metastasized observation.

## Introduction

About one in ten women will at some point develop breast cancer (BC). About 25% have an aggressive cancer at the time of diagnosis, with metastatic spread. The absence or presence of metastatic spread largely determines the patient’s survival. Early detection is hence very important in terms of reducing cancer mortality. A blood sample is cheaper and less invasive than the usual node biopsy. Were we able to detect signs of metastasis or metastatic potential by a blood sample, we could conceivably start treatment earlier.

Several recent articles develop this idea of *liquid biopsies* [1]. A review in *Cancer and Metastasis Reviews* [2] lists liquid biopsies and large data analysis tools as important challenges in metastatic breast cancer research.

The Norwegian Women and Cancer (NOWAC) postgenome cohort [3] is a prospective population-based cohort that contains blood samples from 50,000 women born between 1943 and 1957. Out of these in total about 1600 BC case–control pairs (3200 blood samples) have at various times been processed to provide transcriptomic measurements in the form of mRNA abundance. These measurements combine with questionnaires, disease status from the Norwegian Cancer Registry, and death status from the Cause of Death Registry from Statistics Norway to provide a high-quality dataset. These data are used for exploration and hypothesis generation.

We examine 88 breast cancer cases from the NOWAC study. The blood samples were provided 6–358 days before BC diagnosis. We fit a penalized likelihood logistic regression with the ElasticNet-type penalty [4]. This approach provides built-in variable selection in the estimation procedure. Our model suggests 108 predictor genes that form a potential direction for further research.

## Main text

### Material and methods

#### Data

We analyze 88 cases with breast cancer diagnoses from the NOWAC Post-genome cohort [5]. For each case, we have an age-matched control that we use to normalize the gene expression levels. For our analysis this is mainly done to mitigate batch effects from the lab processing of the blood samples, cases and controls being kept together for the whole pipeline. Only women who received a breast cancer diagnosis at most one year after providing a blood sample were considered as cases. This limits our sample size but it is more biologically plausible to see a signal in more recent blood samples.

Out of the 88 breast cancers, 25% have metastases. The metastic- and non-metastic cancers are fairly similar in terms of usual covariates. Respectively the proportion of smokers is 13% against 25%. The proportion of hormone treatment is 25% against 31%. The median age (with .05 and .95 quantiles) is 56 (51, 61) against 56 (51, 62). The median BMI is 24.5 (19.4, 35.9) against 25.5 (21.1, 32.4). The median parity is 2 (1, 3) against 2 (0, 3).

The data were processed according to [6] and [7]. The pre-processed data is a $88\times 12404$ fold change matrix, *X*, on the $\log _2$ scale. For each gene, *g*, and each observation, *i*, we have the measurement $\log _2 x_{ig} - \log _2 x^\prime _{ig}.$ Here $x_{ig}$ is the *g* expression level for the *i*th case, and $x^\prime _{ig}$ is the corresponding control. The response variable, metastasis, indicates the presence of metastatic spread.

#### Predictive model

We model the probability of metastasis, *p*(*m*), given gene expression across all genes, *x*, by a penalized likelihood logistic regression with an ElasticNet-type penalty [4]. The likelihood of the logistic model

$$\begin{aligned} \log \frac{p(m)}{1-p(m)} = \beta _0 +\beta _1x_1 + \cdots + x_p \end{aligned}$$

is maximized under the constraint that $(1-\alpha )\sum \left| \beta _j\right| + \alpha \sum \beta _j^2 \le t$ for some user-specified penalty size *t* and mixing parameter $\alpha$.

We choose $\alpha =0.5$ a priori and find a penalty size *t* in a data-driven way by optimizing for the modified version of Akaike’s Information Criterion [8, 9],

$$\begin{aligned} AIC^\prime = LR \chi ^2 - 2k, \end{aligned}$$

where $LR \chi ^2$ is the likelihood ratio $\chi ^2$ for the model and *k* is the number of non-zero coefficients. We use this criterion on the recommendation of Harrell [10], who states that maximizing this criterion in terms of penalty often leads to a reasonable choice. We prefer this to tuning by cross-validation since it does not require data splitting. Data splitting procedures tend to induce more variance, which is undesirable with as few observations as we have. A more detailed discussion of these choices can be found in [11].

#### Metrics

We evaluate models by several criteria. Brier score [12] is the mean squared error,

$$\begin{aligned} {{\bar{B}}} = n^{-1} \sum ({\hat{y}}_i - y_i)^2, \end{aligned}$$

between the probability that was predicted by the model, ${\hat{y}}$, and the known outcomes, *y*. It is a one-number summary of the calibration of predicted probabilities.

We also assess calibration by means of a calibration curve. This is an estimate of proportion of true successes as a function of predicted probability, which we calculate by smoothing the true zero/one outcome as a function of predicted probability (LOWESS with a span of $\frac{2}{3}$). If *n* observations receive a prediction of ${\hat{p}}$, $n{\hat{p}}$ of them should have the predicted condition for a well-calibrated model.

Concordance probability is the probability of ranking (in terms of predicted ${\hat{p}}$) a randomly chosen positive higher than a randomly chosen negative. This is equivalent to the area under the receiver operating characteristic curve (AUC), and is proportional to the Mann-Whitney-Wilcoxon U statistic [13].

Stability is the proportion of overlap between predictor genes chosen during different realizations of the modeling procedure. We follow [14] and measure this by the Jaccard index, $\frac{|S_1 \cap S_2|}{|S_1 \cup S_2|},$ where $S_1$ and $S_2$ are two sets of predictor genes.

Brier score and concordance probability are estimated using the optimism-corrected bootstrap approach described in [15], which has the advantage of using all of the data in estimating model performance opposed to data splitting procedures. Stability is estimated from regular bootstrap resampling.

### Results

#### Evaluation metrics

**Fig. 1:**
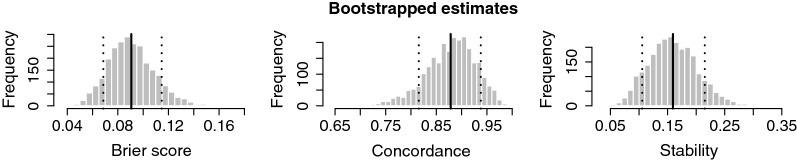
Bootstrap distribution of optimism-corrected estimates for Brier score, concordance/AUC, and stability for the Elasticnet model. The solid vertical lines show point estimates, and the dotted vertical lines show the middle .8 of each distribution

**Fig. 2:**
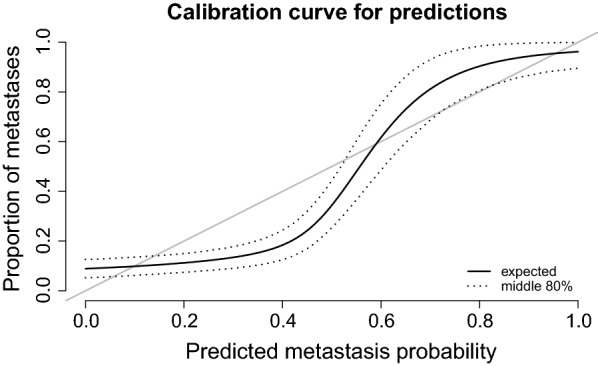
Expected calibration of predicted probabilities shown in solid black. The dotted line shows middle .8 of the bootstrap distribution. Ideally, .8 of the observations for which .8 metastasis probability was predicted should turn out to show metastasis. In other words the ideal calibration is a diagonal line (shown in grey). Our model tends to overestimate lower probabilities and underestimate higher ones

Figure 1 shows the bootstrap distributions for our estimates of Brier score, concordance probability, and stability. The solid lines show point estimates and the dotted lines indicate the middle .8 of each distribution. The Brier score for our model is roughly .1, while that of an intercept-only null model is roughly .18. Since Brier score is the mean square error of predicted probabilities we can take its root to get an average error on the probability scale; $\sqrt{.1} \approx .32$, which suggests that the predicted probabilities are not very accurate on average. Figure 2 corroborates this. The figure shows the pointwise calibration of predicted probabilities, ie., for a given predicted metastasis probability, how great a proportion observations turned out to have metastases. For a predicted metastasis probability $<.4$ the true proportion is $\approx .1$, while for a predicted metastasis probability $>.8$ the true proportion is $\approx .7$. In other words we overestimate low probabilities and underestimate high ones.

Returning to Fig. 1, the concordance probability (or AUC) is quite high at roughly .88, with a lower bound for the middle .8 of the distribution at .81. Contrast this with random guess at .5. This suggests that the model consistently selects gene sets that separate metastases from non-metastases in their expression levels in spite of the fact that the predicted probabilities are poorly calibrated. The stability of these chosen gene sets is around .16, which suggests the likely scenario that there are many correlated genes to choose from. With a stability of .16 for 108 genes you might expect a 17-gene overlap when fitting a similar model to similar data.

#### Selected genes

We list the 108 genes selected by penalized likelihood and describe them in general quantitative terms. We keep track of the selected gene sets under resampling and can hence calculate statistics for how often a given gene is selected and for how often a given gene is co-selected with any other gene. Table 1 shows the 108 selected genes ordered by their individual selection probabilities. Apart from the first few genes, the selection probabilities are not very high. It is quite likely that (i) a larger set of genes correlate with the ones we select and get selected in their place some of the time, and (ii) our selected genes correlate with one another and the selection of one some times makes the selection of another less likely. This is a natural consequence of doing variable selection: “redundant” information may shrink out of the model.

**Table 1:** Resampling selection probability for the 108 elasticnet-selected genes

GRK5^a^	0.853	C1orf115	0.290	ANO8	0.221	FBLN5	0.157
GPATCH4	0.682	LOC654055	0.287	PTTG1IP	0.219	BLMH	0.156
GNGT2	0.474	RNF214	0.280	3NDg8gVCd^b^	0.218	FCRL3	0.149
PDGFD^c^	0.467	SULT1A1	0.278	USF1	0.216	TDRD9	0.143
FAM24B	0.457	ZNF365	0.271	BCCIP	0.210	ACY1	0.142
PTPRN2	0.442	USE1	0.267	MGC29506	0.209	ZFP57	0.142
CBLB	0.440	DNMT3A	0.267	GRK5^a^	0.207	SLIC1	0.138
PDCL	0.410	LOC649210	0.266	WTIP	0.205	PICK1	0.135
RASA2	0.380	CNTNAP2	0.265	BCL10	0.204	RTN4IP1	0.134
C11orf48	0.376	IL2RA	0.265	DLGAP2	0.200	CDCA7L	0.132
TCEB1	0.374	CCT5	0.264	HRAS	0.199	BEX4	0.131
CAPN3	0.354	R3HDM1	0.263	RAD1	0.189	FCAR	0.130
STK19	0.351	MRPL43	0.260	PRKCE	0.187	ANKRD35	0.111
GUCY1A3	0.348	SLC38A1	0.256	UBAP2L	0.186	USP39	0.109
ZDHHC11	0.345	GNG8	0.255	BPI	0.186	KIAA0495	0.106
SULT1A3	0.336	PLA2G4C	0.251	DTX1	0.184	BRI3BP	0.106
Z6FIQGkeo^d^	0.335	TCF4	0.248	LASS5	0.182	TUBA4A	0.105
FAM89A	0.328	uX15cu4f_^e^	0.247	GSTT1	0.182	IDH1	0.102
rh13dQX04^f^	0.324	C20orf107	0.245	SPATA20	0.182	DDX52	0.100
LANCL2	0.323	VCL	0.242	IGLL1	0.172	ANKRD57	0.094
SERPINE2	0.318	EZH2	0.242	SPG3A	0.172	TFG	0.087
ADIPOR2	0.314	PRPSAP2	0.237	PPAP2A	0.172	LILRA6	0.080
GPR177	0.312	ISY1	0.235	NOTCH2NL	0.172	C6orf47	0.078
PDGFD^c^	0.299	UGDH	0.234	TAF6	0.168	WDR60	0.075
LOC647460	0.294	ABCF2	0.230	CCDC90B	0.166	AHCYL2	0.068
WEE1	0.293	C16orf5	0.229	LOC731486	0.158	HAUS4	0.068
ITM2C	0.291	VAV3	0.225	CDH2	0.157	MAD2L2	0.053

^a^ Two probes map to the same gene GRK5. Combined selection probability is 1.06, implying that both get selected together at least some of the time

^b^ Illumina probe id 3NDg8gVCdQkNdcg.Ko, missing annotation

^c^ Two probes map to the same gene PDGFD. Combined selection probability is 0.766

^d^ Ilummina probe id Z6FIQGkeoCSiVAoKeg, missing annotation

^e^ Illumina probe id uX15cu4f_VUIuXoST0, missing annotation

^f^ Illumina probe id rh13dQX04hUS7uOpRQ, missing annotation

The selected genes show a clear difference in fold change between metastasized- and non-metastasized BC cases; we refer interested readers to Additional file 1. Further figures and discussion about, as well as pairwise co-selection can be found in [11].

## Limitations

The prospective design of NOWAC yields data prior to the cancer diagnosis, thus allowing to test prediction models on original data corresponding to early-stage cancer. However, there will perforce never be more cases where the blood sample was provided close to diagnosis in this particular study. As the data acquisition technology has changed, there little hope to produce new comparable data outside of NOWAC. Since our data set is small (88 pairs of women for 12404 probes), we expect the success of both variable selection and prediction to be limited.

Concerning variable selection, the set of genes kept in the model is highly unstable under perturbation by resampling, and only a few of them are selected in a meaningful fraction resamples.

Concerning prediction, the AUC is high enough that there is reason for suspicion. The same is the case for Brier score, which is suspiciously low. It is quite likely that the bootstrap corrections for optimism are too. Moreover the bootstrap shows high variability in high dimensions. The calibration curve suggests that the predicted probabilities need to be better calibrated for this model to be useful for prediction in a real setting.

In model selection with small data sets it is recommended to use AUCc, which places a stronger penalty on larger numbers of parameters than the formulation we use [16]. At the same time we overestimate the effective number of parameters by taking *k* as the number of non-zero parameters, which does not take into account the shrinkage on parameter size. This places a larger penalty than necessary on a given model. Since in our case all models lie on the regularization path decided by the penalty size, a stronger/weaker parameter penalty will lead to similar results in terms of selected genes with some additions/omissions as the case may be.

The model we apply does not control for what is considered usual sources of confounding in breast cancer. This is both out of a desire to identify a pre-diagnosic gene signature for metastasis independent of questionnaire data, and from the realization that this would require the estimation of even more coefficients for already-inadequate data. The potential confounding from sources such as smoking and hormone therapy may not be a problem for prediction, but makes interpretation challenging. On the other hand what is considered a source of confounding for breast cancer may or may not be one when comparing breast cancers to one another in terms of metastasis. The explicit way to deal with this would be to derive a causal model to argue from.

This study is exploratory and not validated in external data. It is important that this work be viewed as hypothesis generating.

## Supplementary information

**Additional file 1.** Expression levels of selected genes. This figure shows the expression levels of selected genes ordered by difference in medians between metastasized andnon-metastasized observations.

## Data Availability

The datasets generated and/or analysed during the current study are not publicly available due to restrictions under Norwegian regulations for access to confidential data based on patient consent and Research Ethics terms, but are available from the corresponding author on reasonable request.

## Abbreviations

AUC: Area under the (ROC) curve
BC: Breast cancer
LOWESS: Locally weighted polynomial regression
NOWAC: Norwegian Women and Cancer
ROC: Receiver operating characteristic
