# Supplementary figures and images for: Predicting breast cancer metastasis from whole-blood transcriptomic measurements
Source: BMC Res Notes. 2020 May 20;13:248. doi: 10.1186/s13104-020-05088-0 (PMC7238609; doi:10.1186/s13104-020-05088-0)

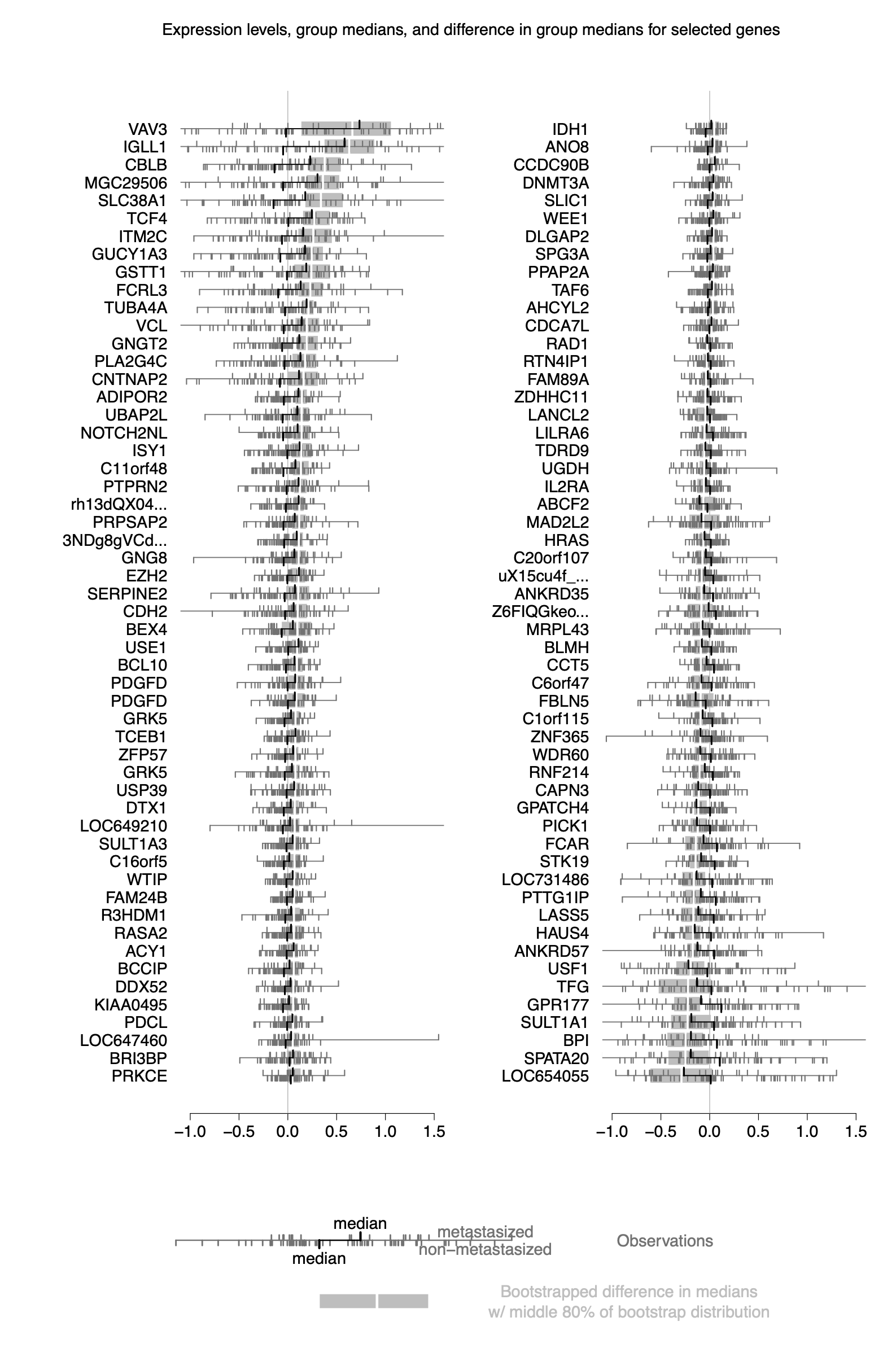

Supplement: Supplementary file 1 — Additional file 1. Expression levels of selected genes. This figure shows the expression levels of selected genes ordered by difference in medians between metastasized andnon-metastasized observations. [file 13104_2020_5088_MOESM1_ESM.png]
